# The archaellum: how Archaea swim

**DOI:** 10.3389/fmicb.2015.00023

**Published:** 2015-01-27

**Authors:** Sonja-Verena Albers, Ken F. Jarrell

**Affiliations:** ^1^Molecular Biology of Archaea, Institute of Biology II-Microbiology, University of Freiburg, Freiburg, Germany; ^2^Molecular Biology of Archaea, Max Planck Institute for Terrestrial Microbiology, Marburg, Germany; ^3^Department of Biomedical and Molecular Sciences, Queen’s University, Kingston, ON, Canada

**Keywords:** archaeal flagellum, archaellum, motility, type IV pili, motor complex

## Abstract

Recent studies on archaeal motility have shown that the archaeal motility structure is unique in several aspects. Although it fulfills the same swimming function as the bacterial flagellum, it is evolutionarily and structurally related to the type IV pilus. This was the basis for the recent proposal to term the archaeal motility structure the “archaellum.” This review illustrates the key findings that led to the realization that the archaellum was a novel motility structure and presents the current knowledge about the structural composition, mechanism of assembly and regulation, and the posttranslational modifications of archaella.

## THE ROAD FROM ARCHAEAL FLAGELLUM TO THE ARCHAELLUM

**FIGURE 1:**
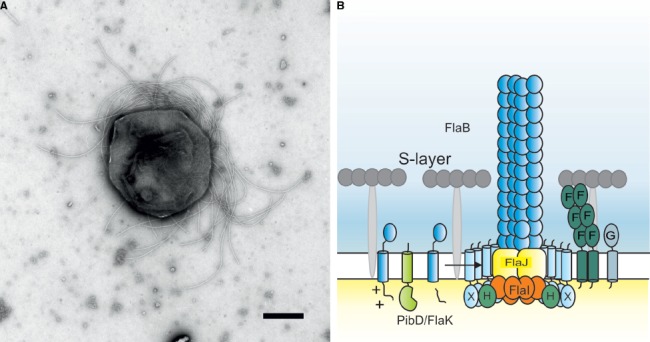
**(A)** Negative stained electron microscopic image of *Methanococcus maripaludis*. Bar length 500 nm. Picture courtesy of S.-I. Aizawa and K. Uchida. **(B)** Current model of the crenarchaeal archaellum. After the pre-archaellin has been processed by PibD/FlaK, the motor complex assembles the filament. The motor complex is formed by the ring-forming scaffold protein FlaX in which FlaH and FlaI interact most probably with the integral membrane protein FlaJ. The dimeric soluble domain of FlaF interacts with the S-layer. FlaG most probably has a similar function as FlaF as it’s soluble domain has homologies to the one from FlaF.

Motility is a trait that is widespread amongst all the different subgroupings of Archaea. While motile archaeal cells possess surface appendages involved in motility that superficially resemble bacterial flagella (Figure 1A), biochemical, genetic, and structural analyses of these archaeal appendages in several model organisms have demonstrated the uniqueness of the archaeal motility structure. This review provides an historical account of the investigations on the archaeal motility structure ending with current studies on the regulation of archaella flagella biosynthesis and determination of the roles of some of the specific components in assembly and function of the organelle.

## EARLY WORK REVEALED UNUSUAL TRAITS OF ARCHAEAL FLAGELLA

The first archaeon to have its flagella studied in detail was *Halobacterium salinarum (halobium)*. Studies by Alam and Oesterhelt (1984) initially revealed several unusual features of the halobacterial flagella. Unlike most bacterial flagella, the flagella of *H. salinarum* form a right-handed helix. Using tethered cells, they showed that these flagella rotate and that the direction of rotation can change from clockwise to counter clockwise (Alam and Oesterhelt, 1984; Marwan et al., 1991). Cells swim forward when the flagellar rotation is clockwise but backward when rotation is counter clockwise. Unlike peritrichously flagellated bacteria, the flagella bundle of *H. salinarum* did not fly apart when rotation direction changed. Flagella were isolated from a “super” flagella overproducer called strain M-175, a strain that shed large numbers of unattached flagella which aggregated into thick bundles containing 100s of individual flagellar filaments. Analysis of these flagella by SDS-PAGE revealed three bands with centers of intensity that corresponded to molecular masses of 26, 30, and 36 kDa, although each of these bands actually consisted of multiple bands in a ladder-like appearance indicating heterogeneity.

This striking pattern revealed by SDS-PAGE was recognized by Wieland et al. (1985) as almost identical to a pattern of heterogeneous sulfated proteins previously studied and thought to be related to bacteriopsin. Their work showed that the flagellin bands reported by Alam and Oesterhelt (1984) were indeed the same as the sulfated proteins. Further study revealed that the flagellins were modified with an N-linked oligosaccharide common to the S layer glycoprotein, the first prokaryotic glycoprotein identified. The N-linked glycan was determined to be Asn-Glc1-4GlcA1-4GlcA1-GlcA and Asn-Glc1-4GlcA1-4GlcA1-4Glc. They studied both the wildtype *H. salinarum* strain and also the superflagella producing M-175 strain and determined that while the pattern was similar in both cases, the entire set of bands was shifted to lower apparent molecular masses in the M-175 strain. It was proposed that the M-175 strain had lost one or more glycosylation sites. Experimental investigation of this proposal was apparently never pursued but subsequent work identifying five flagellin genes (Gerl and Sumper, 1988) makes this explanation unlikely since a loss of a glycosylation site would presumably have to occur in all five flagellins to recreate the observed pattern. It seems more likely that the M-175 strain had a mutation in one of the N-glycan assembly or biosynthesis steps that rendered all five flagellins modified with a truncated glycan and making all the N-glycan-modified proteins migrate as smaller protein on SDS-PAGE. This type of effect was subsequently observed in other archaea like *Methanococcus* species (Chaban et al., 2006; VanDyke et al., 2009), *Haloferax volcanii* (*Hfx. volcanii*; Tripepi et al., 2012), and *Sulfolobus acidocaldarius* (Meyer et al., 2011). Nonetheless, in a prescient hypothesis, Wieland et al. (1985) thought that the overproduction of superflagella by the M-175 mutant could occur if correct glycosylation of the flagellins is necessary for proper incorporation of the flagella into the cell envelope. These were the first prokaryotic flagellins shown to be glycoproteins.

A further key finding was that N-glycosylation in *H. salinarum* occurred on the external surface of the cytoplasmic membrane (Sumper, 1987). This was shown by the addition of ethylenediaminetetraacetic acid (EDTA) which caused a shift in the flagellin molecular masses to the same values as occurs if the flagellins were chemically deglycosylated. In addition, it was shown that an exogenously added peptide carrying an N-glycosylation sequon could be glycosylated even though it could not cross the cytoplasmic membrane. This extracellular site of glycosylation of the flagellins led Gerl and Sumper (1988) to state that “aggregation to a functional flagellum is likely to occur by a mechanism different from that proposed for the assembly of eubacterial flagella.”

**FIGURE 2:**
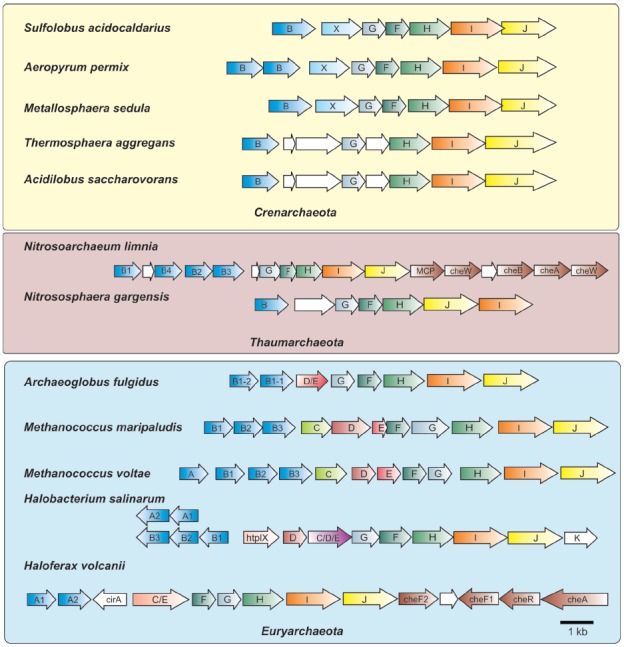
**Organization of archaella operons.** Archaella operons of three of the archaeal kingdoms Crenarchaeota, Thaumarchaeota and Euryarchaeota are depicted. The *fla* genes are abbreviated using the respective letter of the *fla* gene. Homologous genes are shown in the same color. Genes of unknown function are depicted in white. In the strain where chemotaxis genes are adjacent to the archaellum operon they are partially depicted. MCP, methyl accepting chemotaxis protein; *che* genes, genes encoding parts of the chemosensory system; htrl, methyl accepting transducer.

Sumper’s group followed up the glycobiology aspect of the halobacterial flagella with genetic studies. Remarkably, they discovered that *H. salinarum* had five flagellin genes located at two distinct loci in the genome: two genes (*flgA1* and *flgA2*) were located in tandem at one locus while three others (*flgB1*, *flgB2*, and *flgB3*) were found tandemly at a second locus (Figure 2; Gerl and Sumper, 1988). All five flagellin proteins were 193–196 amino acids in length and were remarkably similar in amino acid sequence with large stretches being identical, although there were three short regions of hypervariability that were unique to each flagellin. The calculated molecular masses for all five flagellins were about 20.5 kDa, much smaller than the masses calculated by SDS-PAGE. However, three potential N-linked glycosylation sites were present in each protein. Since the flagellins were already known to be sulfated glycoproteins (Wieland et al., 1985), the heterogeneity seen on SDS-PAGE was explained by the presence of five different proteins which perhaps had different degrees of glycosylation. At the time, a search of protein databanks revealed no significant similarity to other sequences. Critically, the N-terminus of the 26 kDa band was resistant to Edman degradation.

A follow-up study (Gerl et al., 1989) demonstrated that all five of the flagellin proteins could be identified in purified flagella due to the unique amino acid sequences in the variable regions. Such methodology revealed that the flagellins in the 26 kDa band were FlgA2, FlgB1, and FlgB3 while only FlgA1 was found in the 30 kDa band and FlgB2 was the sole flagellin found in the 36 kDa band. Western blotting with specific antibody raised to amino acid sequences unique to the different flagellins also revealed that FlgA1 antisera only reacted to the 30 kDa band and the FlgA2-specific antibodies only reacted to the 26 kDa band.

## DISCOVERY OF SIGNAL PEPTIDES ON ARCHAEAL FLAGELLINS

Flagella were subsequently purified from a number of archaea and the N-terminal amino acid sequence was obtained for a number of these proteins, including one flagellin band from *Methanococcus voltae* (Kalmokoff et al., 1990; Faguy et al., 1994a, 1996). Remarkably, these N-terminal sequences showed no similarity to any bacterial flagellins but all the archaeal sequences showed high amino acid sequence similarity among themselves. Intriguing, the N-terminal sequences obtained aligned with the sequence predicted for the *H. salinarum* flagellin gene sequences but beginning at amino acid position 13, suggesting that the archaeal flagellins were made as preproteins with a signal peptide (Kalmokoff et al., 1990). Shortly thereafter, the flagellin genes of *M. voltae* were cloned and sequence analysis revealed that, indeed, all four flagellin genes of this organism encoded proteins with predicted short signal peptides (Kalmokoff and Jarrell, 1991). This was an unexpected finding since flagellins in bacteria are not made as preproteins and reach their final destination via a flagellum-specific type III secretion system located at the base of the flagellum (Macnab, 2004; Chevance and Hughes, 2008). The flagellins pass through the hollow organelle to the distal tip before incorporation under the flagellar cap protein. Thus, in addition to the unusual structural features reported by Alam and Oesterhelt (1984), archaeal flagella possessed two unique characteristics not found in bacterial flagella: its component subunits were made initially with signal peptides and they were modified with N-linked glycans (Wieland et al., 1985; Kalmokoff and Jarrell, 1991). These two properties suggested a completely novel assembly model was used in archaea for flagella biosynthesis.

## SEQUENCE SIMILARITY OF ARCHAEAL FLAGELLINS TO TYPE IV PILINS AND A NEW MODEL FOR FLAGELLA ASSEMBLY

While initial attempts did not find any relatives of archaeal flagellins in gene databases, Faguy et al. (1994b) reported that the N-terminal region of archaeal flagellins shared sequence similarity to the same highly conserved region in type IV pilins, which themselves formed a different type of appendage on the bacterial cell surface distinct from flagella (Pelicic, 2008; Burrows, 2012). Type IV pilins are known to be made initially as preproteins with unusual signal peptides. The signal peptide is cleaved at a conserved site by a dedicated signal peptidase, termed a prepilin peptidase or signal peptidase III, that is distinct from both signal peptidase I and II (Strom et al., 1994; Lory and Strom, 1997; Giltner et al., 2012). This noted similarity to type IV pilins led to the hypothesis that archaeal flagella could be assembled in a completely novel way compared to bacterial flagella, with insertion of new subunits at the base (Faguy et al., 1994b; Jarrell et al., 1996a). Following the development of the first genetic and transformation systems in *M. voltae* (Gernhardt et al., 1990; Patel et al., 1994), the flagellin genes of this methanogen were targeted and interrupted (Jarrell et al., 1996b). Mutants in the flagellin *flaB2* so generated were non-flagellated, thus linking these genes with the appearance of the flagella on the cell surface for the first time.

## SIMILARITIES OF ARCHAEAL FLAGELLA AND TYPE IV PILI: FURTHER STRUCTURAL AND GENETIC EVIDENCE

Evidence from several avenues of research supporting the notion that the archaeal flagella were distinct from bacterial flagella continued to appear. Electron microscopic examination of purified archaeal flagella revealed a knob at the cell proximal end but no distinct ring structure as seen in flagella of both Gram negative and Gram positive flagella (Kalmokoff et al., 1988; Kupper et al., 1994). Curved hooks regions were observed in some archaeal flagella and specific flagellins were shown to be responsible for this region in both *Methanococcus* and *Halobacterium* (Bardy et al., 2002; Beznosov et al., 2007; Chaban et al., 2007), but this finding was not universal. For example, no hook region has been observed in *Sulfolobus solfataricus*, an archaeon possessing a single flagellin gene (Szabo et al., 2007b). Since most sequenced crenarchaeota genomes only possess a single flagellin gene, the flagella of these organisms would also be expected to lack a hook. Rotation of flagella in *H. salinarum* was shown to be ATP-dependent and not proton motive force (or sodium motive force) driven as it is in bacterial flagella (Streif et al., 2008). Structural studies by the Trachtenberg group revealed further crucial findings. The reconstructed 3D structure of flagella from distantly related archaea (*H. salinarum* and *Sulfolobus shibatae*) was shown to share common features with type IV pili and be distinct from known bacterial flagella structures (Cohen-Krausz and Trachtenberg, 2002, 2008; Trachtenberg and Cohen-Krausz, 2006). Critically, and in support of the type IV pili assembly model proposed earlier by Jarrell et al. (1996a), was the absence of a lumen in the interior of the archaeal flagella that could allow passage of subunits to the distal tip as occurs in bacterial flagella. This seemingly eliminated any potential chance for distal growth of archaeal flagella.

Meanwhile, further genetic evidence emerged that supported the evolutionary relationship of archaeal flagella to type IV pili. Sequencing of genes located downstream of the flagellin genes revealed the presence of two genes that encoded homologues to key components of the type IV pili assembly system, namely a PilB-like polymerizing ATPase (termed FlaI) and the conserved membrane/platform protein (FlaJ; Bayley and Jarrell, 1998; Peabody et al., 2003). Deletion of these genes in various archaea confirmed their involvement in the archaeal flagella system, since these mutants were consistently non-flagellated (Patenge et al., 2001; Thomas et al., 2001b; Chaban et al., 2007; Lassak et al., 2012b). With the advent of the genomic age, many sequenced archaeal genomes were examined and no genes encoding proteins involved in bacterial flagella structure (i.e., rod, hook, rings, etc) were identified (Faguy and Jarrell, 1999; Nutsch et al., 2005; Pyatibratov et al., 2008). Such analyses, as well as directed genetic studies in several archaea, revealed that a conserved group of so-called *fla* accessory genes, often *flaC–flaJ* in euryarchaeotes, was found usually directly downstream of, and co-transcribed with, flagellin genes (in some cases *fla* accessory genes are located in the immediate vicinity but in an opposite orientation to the flagellin genes; see Figure 2; Nagahisa et al., 1999; Patenge et al., 2001; Thomas and Jarrell, 2001; Ng et al., 2006). A typically smaller subset of these genes was observed in the genomes of crenarchaeotes (Ng et al., 2006; Lassak et al., 2012a).

## PROPOSAL TO RENAME THE ARCHAEAL FLAGELLUM AS THE ARCHAELLUM

By 2012, the evidence was overwhelming that there were two distinct flagella structures in the prokaryotic world: the bacterial one and the archaeal one. They were not evolutionarily related and the Archaea domain structure was, in fact, closely related to type IV pili and the homologous type II secretion system which involves a piston-like pseudopilus comprised of pseudopilins and used to push exported proteins through the outer membrane of Gram negative bacteria (Peabody et al., 2003; Korotkov et al., 2012). The sole similarity of the bacterial and archaeal flagella was seemingly in their function as a rotating swimming organelle. With the realization that archaeal flagella were in fact a rotating variant of type IV pili with no evolutionary relationship to bacterial flagella, we proposed that this prokaryotic motility structure be designated the archaellum (Jarrell and Albers, 2012), a distinct name that nevertheless fuses the concept of Archaea and flagellum and thus readily allows for similar terms common in the bacterial flagella field to be used in archaea (i.e., archaella/flagella, archaellins/flagellins, archaellated cells/flagellated cells). This proposal has met with both criticism and support and its acceptance is still under debate in the scientific community (Eichler, 2012; Wirth, 2012), but its use is becoming more common both within the archaeal research community (Stieglmeier et al., 2014; Syutkin et al., 2014) as well as outside the archaeal field (Giltner et al., 2012; Campos et al., 2013). What is undeniable is that each of the three domains of life, Eukarya, Bacteria, and Archaea has entirely distinct “flagella.”

## KEY ENZYME IN ARCHAELLIN PROCESSING: THE PREPILIN PEPTIDASE-LIKE FlaK/PibD

Study of the archaellin signal peptide processing led to the implementation of an assay based on type IV pilin processing to show *in vitro* processing of archaellins that had been heterologously expressed in *Escherichia coli* (Bayley and Jarrell, 1999; Correia and Jarrell, 2000). Shortly thereafter, the gene encoding the prepilin peptidase-like enzyme (FlaK), responsible for processing of the prearchaellins, was identified in both *M. maripaludis* and *M. voltae* and its critical role demonstrated in archaella biosynthesis when deletion of the gene resulted in non-archaellated cells (Bardy and Jarrell, 2002, 2003). Shortly thereafter, a prepilin peptidase-like enzyme, designated PibD, was identified first in *S. solfataricus* and then other archaea that was much broader in its substrate specificity and capable of processing all type IV prepilin-like proteins including archaellins, pilins, and sugar binding proteins (Albers et al., 2003; Tripepi et al., 2010; Henche et al., 2014).

**FIGURE 3:**
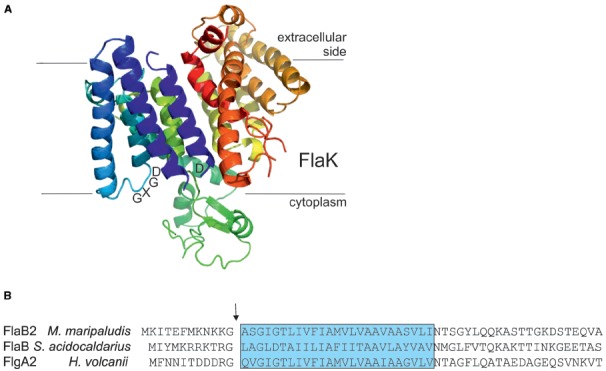
**The archaeal type IV prepilin peptidases. (A)** Crystal structure of FlaK from *M. maripaludis*. Model was constructed with Pymol (www.pymol.org) using the pdb file 3SOX. The GXDG motif is indicated as well as the localization of the membrane. It is clear that the active site is directed toward the cytoplasmic side of the membrane where cleavage of the class III signal peptide occurs. **(B)** The N-terminal archaellin sequences of *M. maripaludis*, *S. acidocaldarius*, and *Hfx. volcanii* are shown. The arrow indicates the cleavage site and the blue box delineates the hydrophobic, membrane inserted part of the mature N-terminus of the proteins. FlaK is specific for the archaellins in *M. maripaludis*, whereas PibD processes a variety of class III signal peptide containing substrates in various archaea.

The archaeal prepilin peptidases FlaK/PibD have both been demonstrated by site-directed mutagenesis studies to belong to the unusual family of aspartic acid proteases that also includes the prepilin peptidases of type IV pili systems in bacteria and presenilin, a protease involved in processing amyloid precursor proteins in humans (LaPointe and Taylor, 2000; Bardy and Jarrell, 2003; Szabo et al., 2006; Ng et al., 2007; Hu et al., 2011; Henche et al., 2014). Unlike the case with prepilin peptidases which methylate the N-terminal amino acid of the processed mature pilins (typically, but not always, a phenylalanine; Strom et al., 1993), the archaeal enzymes have not been shown to possess methyltransferase activity. In these polytopic membrane enzymes, two aspartic acid residues, one located within a conserved classic GxGD motif or a new variant GxHyD [Hy represents a hydrophobic amino acid, most commonly alanine, found in about 60% of archaeal sequenced genomes (Henche et al., 2014)], are critical for the peptidase activity (LaPointe and Taylor, 2000; Bardy and Jarrell, 2003; Szabo et al., 2006; Hu et al., 2011). Recently, the crystal structure of the *M. maripaludis* FlaK was obtained (see Figure 3A; Hu et al., 2011). Analysis of the structure confirmed the presence of six transmembrane helices and demonstrated that FlaK must undergo a conformational change in order to bring the two critical aspartic acid residues, located in transmembrane helix 1 and 4 (the GXGD motif), into close proximity for catalysis.

The typical length of the processed part of the signal peptide on archaellins is 6–12 amino acids (Ng et al., 2006), the short length typical of type IVa prepilins of bacteria (Giltner et al., 2012). In conjunction with studies that investigated the important amino acids in the signal peptidases necessary for catalysis, site-directed mutagenesis studies were also conducted to investigate the importance of various amino acid positions in the signal peptide of archaellins themselves. In the archaellins of *M. voltae*, the highly conserved glycine at the –1 position (position is relative to the cleavage site) was shown to be critical for peptidase cleavage, while the basic amino acids usually found at positions –2 and –3 as well as the conserved +3 glycine also were found to play important roles (Thomas et al., 2001a). Similar studies conducted on the glucose binding protein precursor, used as a model substrate for PibD activity in *S. solfataricus*, indicated PibD was more flexible in accepting amino acid substitutions around the cleavage site than was FlaK, as expected from its broader substrate range (Albers et al., 2003). In *M. maripaludis*, FlaK specifically processes pre-archaellins while the type IV pre-pilins are processed by another type IV prepilin-like peptidase, EppA (Szabo et al., 2007a). *S. solfataricus* PibD can also process the archaellins of *M. voltae* (Ng et al., 2009). In that report, PibD was shown to cleave archaellins engineered with signal peptides as short as 3 and 4 amino acids while for FlaK a minimal signal peptide length of five amino acids was needed for cleavage. This further supports the more flexible nature of the PibD enzyme. Recently, the prepilin peptidase in *Hfx. volcanii*, also designated PibD, was found to be responsible for the processing of both archaellin FlgA2 and other type IV pilin proteins (Tripepi et al., 2010; Esquivel et al., 2013).

A PERL program termed FlaFind, using abundant archaellin sequences available from complete genome sequencing projects as a training set, was developed to predict type IV pilin-like proteins in Archaea based on identification of signal peptides that were similar to those found in archaellins that were known to be processed by archaeal prepilin peptidase-like enzymes (Szabo et al., 2007a). As more experimental evidence accumulated on the actual sequences processed by archaeal signal peptidase III enzymes, a newer version of FlaFind, FlaFind 1.2 (http://signalfind.org/flafind.html), was introduced that allowed for the presence of glutamate and aspartate at the –2 position. The program searches for the conserved signal peptide motif [KRDE][GA][ALIFQMVED][ILMVTAS](Figure 3B; Esquivel et al., 2013).

## BIOCHEMICAL AND STRUCTURAL ANALYSES OF ARCHAELLUM SUBUNITS

In all archaella operons, the genes *flaF,G,H,I,* and *J* are conserved and considered to encode the proteins that form the general assembly machinery and motor complex of this structure (see Figures 1B and 2). All of these genes are essential for archaella assembly and rotation (Patenge et al., 2001; Thomas et al., 2001b; Chaban et al., 2007; Lassak et al., 2012b; Reindl et al., 2013). FlaI was demonstrated to have ATP hydrolyzing activity, which was greatly stimulated by the addition of archaeal lipids (Albers and Driessen, 2005; Ghosh et al., 2011). FlaI forms an ATP-dependent hexamer and was crystallized in different nucleotide-bound states (Ghosh et al., 2011; Reindl et al., 2013). The C-terminal domain (CTD) of FlaI, which contains the Walker A and B motif for ATP-binding and hydrolysis, interacts more strongly with the N-terminal domain (NTD) of the neighboring monomer than with its own NTD. It is hypothesized that this strong interaction is essential for the function of FlaI in the rotation of the archaellum filament.

In the FlaI hexamer, the N-termini of each monomer form the tips of the crown-like complex. In contrast to the nucleotide-free FlaI hexamer, the tips of the crown were rotated in a perpendicular fashion inside the hexamer in the nucleotide bound state. It is proposed that the tips of FlaI lock into the cytoplasmic loops of FlaJ, the only polytopic membrane protein of the archaellum machinery, and thereby form a rigid motor complex to drive rotation of the archaellum filament (Reindl et al., 2013).

Another subunit of the archaellum, which was biochemically and structurally analyzed, is FlaX. While FlaX is essential for archaellation in *S. acidocaldarius* (Lassak et al., 2012b), it is not found in euryarchaeotes. FlaX is a monotopic membrane protein and its soluble domain was shown to form large oligomeric ring structures of around 30 nm diameter (Banerjee et al., 2012). It was shown that the coiled-coil region that is present in the middle of its soluble domain is essential for FlaX ring formation. Both parts of FlaI, the N- and the C-terminus, were shown to interact with the soluble part of FlaX (Banerjee et al., 2013).

In addition to FlaI, FlaH is the only other predicted cytoplasmic component of the archaellum assembly machinery. Although FlaH exhibits a Walker A motif, its non-canonical Walker B motif suggests that FlaH is not an active ATPase. It is proposed that it might modulate the activity of FlaI. A structure of *Pyrococcus horikoshii* FlaH (PH0284) is present in the Protein Data Bank, but has not yet been described. It shows high similarity to RecA folds, but no nucleotide was present in the structure. Using different biochemical assays, it was demonstrated that FlaX, FlaI, and FlaH indeed form a stable complex (Banerjee et al., 2013), which is thought to anchor the cytoplasmic part of the motor complex of the archaellum. The binding affinities of the single subunits to each other were all in the nanomolar range.

FlaF and FlaG are also conserved components of the archaellum assembly machine. Their order in archaella operons, however, clearly sets euryarchaea apart from crenarchaea (Desmond et al., 2007). Both FlaF and FlaG are monotopic membrane proteins. FlaF contains a partial predicted archaellin domain implying that its soluble domain might be located in the pseudo-periplasm. Very recently, the crystal structure of FlaF from *S. acidocaldarius* was solved (Banerjee et al., submitted). It revealed a β-sheet-dominated structure with homologies to immunoglobulin folds and the recently solved structure of SbsB, the S-layer protein of *Geobacillus stearothermophilus* (Baranova et al., 2012). Binding assays with isolated *S. acidocaldarius* S-layer showed that FlaF bound to the S-layer, implying that it might be involved in anchoring the archaellum in the archaeal cell envelope (Banerjee et al., submitted). It was shown that dimerization is important for FlaF’s function and therefore it is proposed that FlaF forms a channel between the cytoplasmic membrane and the S-layer in which the archaellum filament can cross the pseudo-periplasmic space and the S-layer. A current model of the crenarchaeal archaellum is depicted in Figure 1B.

## THE ARCHAELLUM IS A ROTATING TYPE IV PILUS

When Alam and Oesterhelt (1984) showed that the archaella of *H. salinarum* were rotating, this was, at first sight, not surprising as they were being compared to bacterial flagella which were known to behave similarly. Later, *Sulfolobus* cells were also observed to rotate when tethered to a surface (Grogan, 1989). However, in light of the archaeal motility structure subsequently being identified as a type IV pilus structure, this rotation feature became exciting again. Type IVa pili are known to be extended and retracted by the action of two ATPases, PilB, and PilT, respectively (Merz et al., 2000). This feature enables bacteria to move across surfaces in a process termed twitching. The bacterium is pulled over a surface when extended pili adhere to the surface and subsequently retract (Burrows, 2005). However, type IV pili have not been reported to rotate, although a model was recently proposed in which the pseudopilus of a type II secretion system rotates during its assembly (Nivaskumar et al., 2014). While *H. salinarum* was shown to be able to switch the rotation direction of its archaella depending on different light pulses (Alam and Oesterhelt, 1984), the switching of the *Sulfolobus* archaellum seems to be a stochastic event (Shahapure et al., 2014). 72% of tethered *S. acidocaldarius* cells were found to be rotating counterclockwise, whereas 10% were switching spontaneously, and 18% of the cells were spinning clockwise. The archaellum switching events in *H. salinarum* are governed by the action of the chemotaxis/phototaxis system which has been studied in detail in this organism (Marwan et al., 1990; Rudolph et al., 1995, 1996; Rudolph and Oesterhelt, 1996; Schlesner et al., 2012). Many facets of the chemotaxis systems of bacteria and archaea seem to be conserved (Szurmant and Ordal, 2004) but it remains to be elucidated how the chemotaxis system can enact switching events in two absolutely different motility structures, the flagellum and the archaellum. For the archaellum, data on this topic is extremely limited. Schlesner et al. (2009) identified three proteins in *H. salinarum* that interacted with both chemotaxis proteins and the archaella proteins FlaCE and FlaD. Two of these proteins belong to protein family DUF439 while the third is a HEAT_PBS family protein. Deletion of one of the DUF439 proteins or the HEAT_PBS family protein led to cells that could not switch the direction of archaella rotation. These proteins provide a link between the signal transduction of the chemotaxis system and the archaella.

## KEY ROLE FOR N-LINKED GLYCOSYLATION IN ARCHAEAL FLAGELLA ASSEMBLY AND FUNCTION

With the availability of complete genome sequences for many archaellated archaea and the development of genetic techniques for generating targeted gene deletions, advances were made in the analysis and importance of the N-linked glycosylation found on the archaellins in model archaea (Gernhardt et al., 1990; Tumbula et al., 1994; Moore and Leigh, 2005; Leigh et al., 2011; Wagner et al., 2012; Jarrell et al., 2014). This work was originally performed on *Methanococcus* species where either a trisaccharide (*M. voltae*; Voisin et al., 2005) or tetrasaccharide (*M. maripaludis*; Kelly et al., 2009) glycan was found linked to each of the multiple archaellins that comprise the archaellum filament. It was quickly observed that deletion of *aglB* (the oligosaccharyltransferase responsible for transfer of the completed glycan from its dolichol lipid carrier onto the target protein) resulted in non-archaellated cells, suggesting that the archaellins must undergo the N-glycosylation modification to be properly incorporated into a filament on the cell surface [Chaban et al., 2006; VanDyke et al., 2009; as considered earlier for *Halobacterium* M175 (Wieland et al., 1985)]. Further studies demonstrated that mutants carrying deletions in other *agl* (*a*rchaeal *gl*ycosylation) genes involved in either biosynthesis of the individual sugars of the glycan or its assembly on the lipid carrier (various glycosyltransferases) also led to defects in either archaellum assembly or motility (VanDyke et al., 2008, 2009; Chaban et al., 2009; Jones et al., 2012). In the case of both *Methanococcus* species, synthesis of a glycan of at least two sugars was necessary in order for cells to be archaellated. In the case of *M. maripaludis*, motility was correlated directly with the size of the glycan with wildtype cells carrying the tetrasaccharide glycan being more motile than cells carrying archaellins with a trisaccharide glycan which in turn were more motile than cells carrying archaellins modified with a disaccharide (VanDyke et al., 2009). Similar observations were also reported in both *S. acidocaldarius* and *Hfx. volcanii* where studies on N-linked glycosylation, initially focused on effects on the S-layer protein (Eichler, 2013; Kaminski et al., 2013; Meyer and Albers, 2013), turned also to an examination of this posttranslational modification on surface appendages. Again interference in the N-glycosylation pathway had major effects on archaellation and motility. In *Hfx volcanii*, where archaellins are decorated with a pentasaccharide, mutants deleted for *aglB* were non-archaellated (Tripepi et al., 2012). Investigations with strains deleted for other *agl* genes indicated that likely a minimum three sugar glycan was necessary for proper archaella formation and/or function. Site directed mutagenesis to remove each of the three N-glycosylation sites of archaellin FlgA indicated that modification at all sites was necessary for archaella formation. In *S. acidocaldarius*, recent evidence also showed that interference in the N-glycosylation system also led to non-archaellated cells (Meyer et al., 2011, 2013). However, it could be demonstrated in this organism that it was not the glycosylation of the archaellin itself that is important for archaella stability, but rather the N-glycosylation pathway is probably essential for archaella assembly. Deletion of five of the six N-glycosylation sites of the lone archaellin led to no decrease in motility, whereas the deletion of genes of the N-glycosylation pathway did. Therefore, it was proposed that the correct N-glycosylation of cell wall components plays an important role in archaella assembly (Meyer et al., 2014). Interestingly, in *M. maripaludis*, elimination of the four N-glycosylation sites in all possible combinations in one of the major archaellins, FlaB2, indicated that archaella could be assembled and function if FlaB2 was missing three of the four sites but not all of them (Ding et al., in press). Thus, it seems, that depending on which model organisms is being studied, N-glycosylation of the archaellins may be necessary at all N-glycosylation sites (*Hfx. volcanii*), at none of the sites (*S. acidocaldarius*) or at some of the sites (*M. maripaludis*) for archaella assembly.

## REGULATION OF ARCHAELLA COMPONENT EXPRESSION

The regulation of the archaellum operon is, so far, restricted to a few examples. In studied methanogens, biosynthesis of archaella is not constitutive: it is known in both *Methanocaldococcus jannaschii* and *M. maripaludis*, for example, that archaella synthesis is induced under H_2_ limitation conditions (Mukhopadhyay et al., 2000; Hendrickson et al., 2008). Quantitative proteomics of nutrient-limited *M. maripaludis* further demonstrated that the expression of archaellins was affected by multiple nutritional factors: decreased expression was observed under nitrogen limitation but increased expression when cells were phosphate limited (Xia et al., 2009). To date, no transcriptional regulators involved in archaellation have been identified in any euryarchaeon.

**FIGURE 4:**
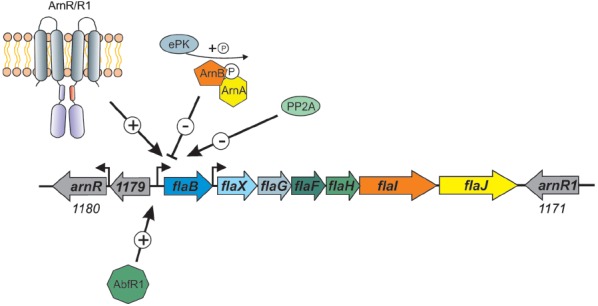
**Overview of the factors that influence the expression of the *S. acidocaldarius* archaellum operon.** The one component membrane factors ArnR/R1, as well as the biofilm regulator AbfR1, are positive regulators of the *flaB* promoter. The two kinases ArnC and ArnD (Saci_1193 and Saci_1694, respectively), here depicted as ePK (eukaryotic like protein kinase) phosphorylate ArnA and ArnB which leads to repression of flaB expression. Deletion mutants of the phosphatase PP2A have a hypermotile phenotype, however, the specific target of the phosphatase is not known.

However, it is in the crenarchaea that most of the information concerning regulation of archaella is known. It was demonstrated in *S. solfataricus* that starvation induced the expression of the archaellum operon (Szabo et al., 2007b). In *S. acidocaldarius*, a number of components of the archaellum regulatory network (termed Arn proteins) were identified. ArnA, containing a fork head associated (FHA) domain and a zinc finger domain, was first shown in *S. tokodaii* (Wang et al., 2010) to be phosphorylated by kinase ST1565. A screen with *S. tokodaii* promoters identified the *flaX* promoter as a target, which was only bound when ArnA was in the phosphorylated state (Duan and He, 2011). ArnA is co-transcribed in an operon with ArnB, which contains a van Willebrand domain. These two proteins were demonstrated to strongly interact with each other both *in vitro* and *in vivo* in *S. acidocaldarius* (Reimann et al., 2012). As FHA domain containing proteins are known to bind to phosphorylated tyrosines, it is proposed that the ArnA and ArnB interaction relies on protein phosphorylation. Deletion of ArnA, ArnB or the zinc finger of ArnA led to the overexpression of archaella in *S. acidocaldarius* even without starvation conditions, indicating that both proteins act as repressors of the archaellum operon (see Figure 4; Reimann et al., 2012). In the *fla* operons of Sulfolobales, three other conserved proteins were identified, Saci_1180 (ArnR), Saci_1171 (ArnR1) and Saci_1179. Saci_1179 is a small membrane protein; deletion of the corresponding gene did not lead to any deregulation of archaella in *S. acidocaldarius* (Lassak et al., 2013). On the contrary, deletion of Saci_1180 completely inhibited expression of FlaB (Lassak et al., 2013). Saci_1180 is a membrane bound one-component regulator, termed ArnR, with an N-terminal helix trun helix (HTH) domain and two C-terminal transmembrane domains (Figure 4). In between these two domains a possible sensing domain is present which is believed to transmit a signal to the HTH domain. Interestingly, only in *S. acidocaldarius*, a gene duplication has occurred as downstream of *flaJ*, an *arnR* paralog is present, termed *arnR1* (see Figure 4). The HTH domains of ArnR and ArnR1 are nearly identical, whereas their sensing domains are quite different. Deletion of ArnR1 had a much less severe effect on *flaB* expression, indicating that it might be involved in fine tuning the expression of *flaB*. The archaellum operon in *S. acidocaldarius* has two transcriptional units of which one is *flaB* and the other locus is *flaX-J* (see Figure 4; Lassak et al., 2012b). Promoter fusion assays showed that ArnR and ArnR1 regulate the *flaB* promoter but not the *flaX-J* promoter. Moreover two inverted repeats, which are essential for the transcription of *flaB*, were identified in the promoter region of *flaB* (Lassak et al., 2012b).

The activity of members of the crenarchaeal archaellum regulatory network is regulated by protein phosphorylation. This was shown first for ArnA from *S. tokodaii* (Wang et al., 2010), then ArnA and ArnB were demonstrated to be phosphorylated by the protein kinase Saci_1193 (Reimann et al., 2012), now termed ArnC and only ArnB was phosphorylated by Saci-1694 in *S. acidocaldarius* (ArnD; Reimann et al., 2012). Moreover, in a phosphoproteomic study, the deletion of PP2A, the serine/threonine phosphatase of *S. acidocaldarius*, led to a strong overexpression of all archaella genes, whereas the deletion of protein tyrosine phosphatase (PTP), the tyrosine phosphatase, had no effect on archaella expression (Figure 4; Reimann et al., 2013).

Another regulator, AbfR1 (archaeal biofilm regulator 1) was also demonstrated to be involved in the archaellum regulatory network in *S. acidocaldarius* (Orell et al., 2013b). AbfR1 belongs to the Lrs14 regulator family of which two other members are also implicated in the regulation of biofilm growth. In the AbfR1 deletion mutant, the synthesis of archaellum components was impaired (Figure 4), leading to an increased production of EPS and biofilm (Orell et al., 2013a,b). In different archaea, the expression of other type IV pili also seems to influence the expression of archaella. In *S. acidocaldarius*, the deletion of the gene encoding the membrane protein AapF from the archaeal adhesive pili operon unexpectedly led to a strong induction of archaella, indicating that a switch exists that determines which of the surface structures is expressed (Henche et al., 2012). In *Hfx. volcanii*, it was recently observed that the deletion of *flgA2*, encoding the second archaellin in this organism, led to hypermotile cells with an increased number of archaella (Tripepi et al., 2013). Moreover, the presence of the H-domain of a set of type IV pilins (PilA1-A6) post-translationally influenced the assembly of archaella in *Hfx. volcanii* (Esquivel and Pohlschroder, 2014). When the pilins were deleted, the cells were non-motile whereas the deletion of the pilus assembly machinery had no influence on archaella assembly implying that the presence of the pilin subunits in the membrane is important for the regulation of archaellum assembly in *Hfx. volcanii*.

In *Haloarcula marismortui*, the two different archaellins FlaA2 and FlaB were produced under different growth conditions (Syutkin et al., 2014). Archaella assembled from FlaA2 were more stable than archaella built from FlaB and, therefore, they were called ecoparalogs as they were produced under different environmental conditions.

## CONCLUSION

During the last few years, an increasing amount of evidence has been collected proving that the archaeal motility structure is structurally and evolutionarily unrelated to the bacterial flagellum, leading directly to the proposal to rename the structure as the archaellum. Although work on the regulation and the assembly of the archaellum has been initiated, we still do not understand how this quite simple motor can achieve power comparable to that generated by the bacterial flagellum. Indeed, the recently measured swimming speeds of several hyperthermophilic archaea at 400–500 body lengths per second, clearly indicate that these organisms can be considered the fastest on Earth, all powered by archaella (Herzog and Wirth, 2012). Future work will no doubt concentrate on this intriguing aspect of this unusual prokaryotic organelle as one major research focus even as efforts are also made to understand the regulation of the assembly of the structure and the critical role that the N-glycosylation pathway plays.

### Conflict of Interest Statement

The authors declare that the research was conducted in the absence of any commercial or financial relationships that could be construed as a potential conflict of interest.
